# Recurrent Microdeletions at Xq27.3-Xq28 and Male Infertility: A Study in the Czech Population

**DOI:** 10.1371/journal.pone.0156102

**Published:** 2016-06-03

**Authors:** Blanka Chylíková, Ivan Hrdlička, Kamila Veselá, Karel Řežábek, František Liška

**Affiliations:** 1 Institute of Biology and Medical Genetics, 1st Faculty of Medicine, Charles University in Prague and General University Hospital, Praha, Czech Republic; 2 Center for Assisted Reproduction, Clinic of Gynecology and Obstetrics, 1st Faculty of Medicine, Charles University in Prague and General University Hospital, Praha, Czech Republic; Clermont-Ferrand Univ., FRANCE

## Abstract

**Background:**

Genetic causes of male infertility are hypothesized to involve multiple types of mutations, from single gene defects to complex chromosome rearrangements. Recently, several recurrent X-chromosome microdeletions (located in subtelomeric region of the long arm) were reported to be associated with male infertility in Spanish and Italian males. The aim of our study was to test their prevalence and infertility association in population of men from the Czech Republic.

**Methods:**

107 males with pathological sperm evaluation resulting in nonobstructive infertility were compared to 131 males with normal fecundity. X-chromosome microdeletions were assessed by +/- PCR with three primer pairs for each region Xcnv64 (Xq27.3), Xcnv67 (Xq28) and Xcnv69 (Xq28). The latter microdeletion was further characterized by amplification across the deleted region, dividing the deletion into three types; A, B and C.

**Results:**

We detected presence of isolated Xcnv64 deletion in 3 patients and 14 controls, and Xcnv69 in 3 patients and 6 controls (1 and 1 patient vs.4 and 1 control for types A and B respectively). There was one control with combined Xcnv64 and Xcnv69 type B deletions, and one patient with combination of Xcnv64 and Xcnv69 type C deletions. The frequency of the deletions was thus not higher in patient compared to control group, Xcnv64 was marginally associated with controls (adjusted Fisher´s exact test P = 0.043), Xcnv69 was not associated (P = 0.452). We excluded presence of more extensive rearrangements in two subjects with combined Xcnv64 and Xcnv69 deletions. There was no Xcnv67 deletion in our cohort.

**Conclusion:**

In conclusion, the two previously reported X-linked microdeletions (Xcnv64 and Xcnv69) do not seem to confer a significant risk to impaired spermatogenesis in the Czech population. The potential clinical role of the previously reported patient-specific Xcnv67 remains to be determined in a larger study population.

## Introduction

Infertility is a widespread health problem. According to a global meta-analysis comprising 172413 women, 9% (i.e. about 70 million) of couples are infertile worldwide [1]. Another recent metaanalysis estimated male infertility prevalence in Central and Eastern Europe to 8–12% [2] mainly based on data from Poland [3]. Male factor is responsible for about half of the cases [2]. Substantial proportion of male infertility cases remain unexplained despite advances in clinical diagnostics; a large proportion of the latter is thought to be genetic in origin [4, 5].

Most prevalent genetic cause of male infertility is Klinefelter syndrome, about 4% in unselected infertile males [6, 7] and the azoospermia factor (AZF) deletions in the male specific region of the Y chromosome, ranging from 4% in oligospermic males to 11% in azoospermic males [8]. Major cause of genetically originated obstructive azoospermia is homozygous or compound heterozygous mutation of cystic fibrosis transmembrane regulator (CFTR), due to missing or obstruction of seminiferous ducts [9, 10].

Unprecedented progress in human genetics and genomics is leading to identification of genes that are the cause of human infertility [10–12]. However, mutations in these genes are relatively rare, and can explain only a small subset of male infertility causes. Moreover, there is still a large gap between the number of genes that are known to play a role in male fertility in the mouse (947 genotypes in mouse phenotype browser at http://www.informatics.jax.org/searches/MP_form.shtml for “MP:0001925—male infertility” in mouse genome informatics database [13]) or other animal models, and the number of genes identified to play this role in human males.

Recently, several recurrent X-chromosome microdeletions (located in subtelomeric region of the long arm) were reported to be associated with male infertility in Spanish and Italian males [14]. The authors found significant association of three microdeletions with male infertility: Xcnv64 (cytogenetic location Xq27.3), Xcnv67 (Xq28) and Xcnv69 (Xq28). The microdeletion Xcnv67 was detected exclusively in infertile men. The aim of our study was to test their prevalence and infertility association in men with unexplained infertility from the Czech Republic.

## Materials and Methods

### Subjects

The study was approved by Ethics Committee of General University Hospital in Prague; all subjects consented in written form to use of their genomic DNA for research in genetics of male infertility. Subjects were divided in two groups. For patient group, we used DNA samples that were collected for subjects who sought genetic counselling because of infertility and who presented with abnormal results of semen analysis. For control group, we endorsed men who were counselled as potential sperm donors, or were counselled as healthy family members in families suffering from genetic disorders distinct from infertility.

Limiting the subjects to Czech ethnicity by self-declaration, we initially collected 154 individuals that were undergoing genetic consultation regarding male infertility and who presented with abnormal semen analysis. Although the subjects who all underwent genetic counselling should be already preselected for genetic causes of infertility, we still excluded men with accompanying traits that could potentially cause or aggravate infertility without genetic contribution. Thus we excluded subjects with varicocele (18), since varicocele can be a sole cause of infertility, albeit in a minority of cases [15];.bilateral or late operated cryptorchidism (2); state after uroinfection/epididymal obstruction (4); state after chemotherapy for cancer (2); cyst of testes (1) and hormonal dysbalances (low testosterone or high prolactin levels, 3).

Based on routine genetic evaluation we also excluded subjects with unbalanced karyotype (9 patients with Klinefelter syndrome, one patient with 47, XYY karyotype, and one patient with a marker chromosome), with Y-chromosome deletions–AZFa (1), AZFb (1, this person had also varicocele) and AZFc (4), and one subject who was a compound heterozygote for CFTR mutations. Before exclusion, frequency of Klinefelter syndrome in our putatively nonenvironmental, i.e. genetic male infertility patients was 7.2% (9/125), frequency of AZF deletions was 4.8% (6/125), and frequency of CFTR-related infertility was 0.8% (1/125). These frequencies were comparable to other studies (e.g. [16], see also the references in introduction). The final cohort consisted of 107 subjects with male infertility, likely with contribution of other unrecognized genetic factors.

Controls comprised 131 subjects with normal spermiogram, most often sperm donors or men who fathered children without the help of assisted reproduction techniques.

### Sperm evaluation

Semen samples were obtained by masturbation. Sperm analyses were performed according to the WHO laboratory manual for the Examination and processing of human semen (fifth edition, 2010). We performed macroscopic examination (liquefaction, semen viscosity, appearance of the ejaculate, semen volume, semen pH) and microscopic investigation (aggregation or agglutination of spermatozoa and cellular elements other than spermatozoa). Next we did evaluation sperm concentration, motility, percentages of progressively motile and number of morphologically normal spermatozoa. We used Makler chamber for counting of spermatozoa. Semen parameters were classified as follows: oligospermia = total sperm concentration < 15 millions/ml, teratozoospermia = morphology < 4% normal forms, asthenozoospermia = progressive motility < 32%. Cryptozoospermia = total sperm concentration < 1000/ml.

### Genotyping

Genomic DNA was isolated from peripheral blood using QIAamp DNA Mini Kit (Qiagen) according to manufacturer´s instructions.

Primers for amplification of genomic DNA in the potentially deleted regions Xcnv64 (Xq27.3), Xcnv67 (Xq28) and Xcnv69 (Xq28) were adopted from [14]. Briefly, one primer pair served to identify the deletion (amplification negative), two other primer pairs upstream and downstream in the deleted region were used to confirm the deletion. We used Taq DNA polymerase (ThermoFisher Scientific) and 60°C annealing for the whole cohort. In individuals that appeared to carry the deletion, we tested lower annealing temperatures (down to 53°C) and also high-processivity and high-fidelity Phusion DNA polymerase (Finnzymes) to control for falsely negative amplification in case of suboptimal DNA quality. To further classify the Xcnv69 deletion, primers flanking the deletion breakpoints were used to amplify across the deleted segment. Type A and type B deletions are defined by positive amplification using the respective A or B specific primers; type C is negative for both reactions [14]. To confirm the breakpoints, the PCR products were sequenced using BigDye Terminator v1.1 Cycle Sequencing Kit (Applied Biosystems).

### Statistical analysis

Statistical analysis was performed using Statistica 12. Differences in age, weight, height, sperm concentration etc., were assayed using Student´s t-test for independent samples. Significance of the chromosome X microdeletions association with infertility was assayed using two-tailed Fisher exact test. Holm-Bonferroni method was used for multiple comparison adjustment. Null hypothesis was rejected whenever P < 0.05.

## Results

### Patient and control groups

The patient group consisted of 107 subjects. According to sperm evaluation, 23 subjects were azoospermic. The remaining 84 were diagnosed to have oligoasthenozoospermia (57), oligoasthenoteratozoospermia (16), oligozoospermia (10), isolated asthenozoospermia (3) and cryptozoospermia (1). We compared the group to the 131 subjects of normal spermiogram and/or proven natural fertility. There was no significant difference in age at examination, height and weight (including body mass index) of the infertile males compared to controls (Table 1). There was higher smoking prevalence in the infertile group; however, in smokers, there was no difference in cigarette consumption (S1 Table). Chronic prescription drug use and concomitant chronic illness (of any type that is not directly linked to infertility, like hypertension, allergy, depression etc.) were not more common in the infertile patients compared to controls (8 vs. 14, Fisher´s exact P = 0.502 and 30 vs. 49, P = 0.131.respectively).

**Table 1:** Basic biometric data of the studied population sample.

Trait	infertile men (n = 107)	controls (n = 131)	p
Age at examination (years)	34.8 ± 5.2	34.3 ± 5.8	0.46
Height (cm)	181.7 ± 7.2	181.5 ± 7.5	0.85
Body weight, kg	91.5 ± 14.9	88.5 ± 14.2	0.13
BMI (kg.m^-2^)	27.7 ± 3.8	26.9 ± 3.9	0.13

Values are shown as mean ± standard deviation. P-values are given for unpaired Student's t-test.; BMI = body mass index.

### Prevalence of Xq microdeletions

**Fig 1:**
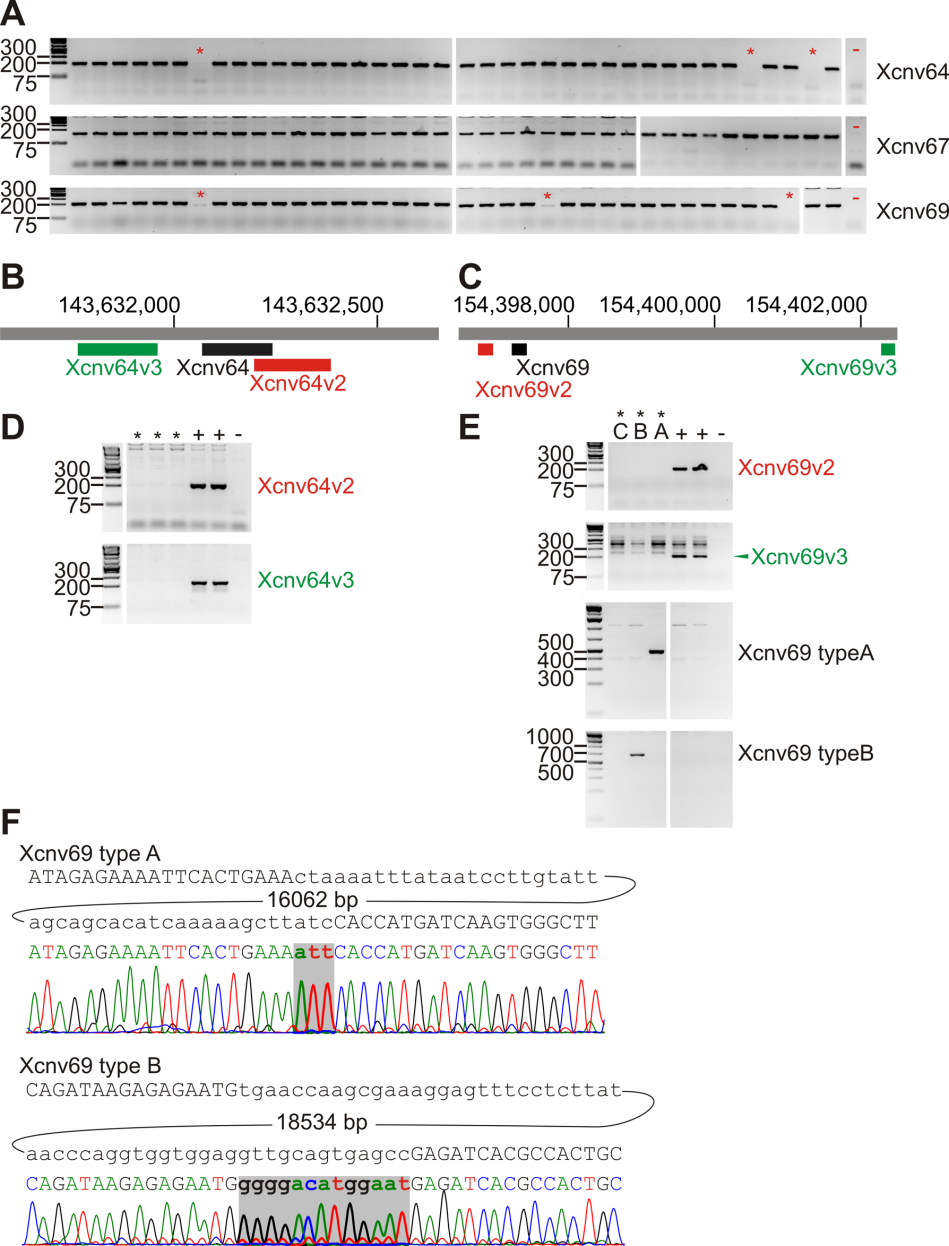
Detection of chromosome X microdeletions by PCR. (A) Electrophoresis of PCR products amplified using primers situated in the regions of Xcnv64, Xcnv67 and Xcnv69 deletions in a portion of the study population. Samples with deletion are marked by asterisks. “-”represents negative (no DNA, water only) controls. (B) and (C) Localization of the amplicons used to capture (black) and confirm (colored) the Xcnv64 and Xcnv69 deletions, respectively. Base numbering according to hg19. (D) Confirmation of Xcnv64 deletion in samples detected as carrying the deletion in A (asterisks). Positive controls (samples without deletion) are labeled “+”, negative control “-“. (E) Confirmation of Xcnv69 deletion in samples detected as carrying the deletion in A (asterisks). In addition to +/- PCR, amplification of the deletion breakpoints was carried out to distinguish types A, B and C of Xcnv69. Positive and negative controls are labeled as in D. (F). Sequencing of deletion breakpoint amplicons from patients with Xcnv69 type A and type B respectively. Above each electropherogram is the reference sequence at the breakpoint with the size of intervening DNA segment (bp). Bases inserted at the breakpoint are in bold. Other samples with the same type of deletion were identical to the displayed representative sequences.

We assayed the previously identified microdeletions Xcnv64 (Xq27.3), Xcnv67 (Xq28) and Xcnv69 (Xq28) by PCR amplification of a fragment inside the deleted regions, and confirmed the deletions by amplification of different fragments upstream and downstream in the deleted region (Fig 1A–1E). We detected presence of Xcnv64 in 3 patients and 14 controls. The difference reached statistical significance marginally (multiple testing corrected Fisher´s exact test p = 0.0432), for higher incidence in controls, in contrast to the South European cohort. We detected Xcnv69 in 3 patients and 6 controls, which does not suggest an association between male infertility and Xcnv69 deletion (p = 0.452). Xcnv69 deletions can be divided in type A + B that can be detected by amplification of fragment across the deleted regions and type C that fails to be amplified in this way (Fig 1E and 1F). Thus, 1 patient and 4 controls had type A deletion and one patient together with one control had type B deletion (Table 2). In addition, one control had type B deletion in Xcnv69 and Xcnv64 deletion and one infertile subject showed type C deletion in Xcnv69 in combination with Xcnv64 deletion. In the last case, there was a possibility, that a more extensive rearrangement was present with putative causal potential; however, markers of the intervening segment were present (Table 3). Therefore, inversion could be the only nonexcluded rearrangement comprising both Xcnv64 and Xcnv69 deletions. There was no clear difference in the sperm parameters of the men with the microdeletions compared to the men without the microdeletions (Table 4), with the exception of progressive motility (%) and total motile sperm count, which were marginally lower in the group of fertile normospermic men with an X chromosome microdeletion compared to fertile normospermic men without any microleletions (Table 4). Results of sperm evaluation for all individuals with X chromosome microdeletions are given in S2 Table.

**Table 2:** Xq27-Xq28 microdeletions detected among the infertile males and controls.

Trait	infertile men	controls	P(raw)	P(corr)
Xcnv64	3	14	0.0216	0.0432
Xcnv67	0	0	N.D.	N.D.
Xcnv69 type A	1	4	0.452^a^	0.452^a^
Xcnv69 type B	1	1		
Xcnv69 type C	0	0		
Xcnv64+69B	0	1		
Xcnv64+69C	1	0		
no deletion	101	111		
sum	107	131		

P(raw) represent P-values of Fisher´s exact test, P(corr) value is multiple comparison corrected using Holm-Bonferroni method.

^a^ These P-values were calculated using all three types of Xcnv69 deletion.

**Table 3:** Sequence tagged sites (STSs) in vicinity of the reported X-chromosome microdeletions.

	143,624	143,632 (Xcnv64)	143,640	148,650 (Xcnv67)	154,366	154,397 (Xcnv69)	154,417
Xcnv64 (all)	+	-	+	+	+	+	+
Xcnv69A (all)	+	+	+	+	+	-	-
Xcnv69B (all)	+	+	+	+	+	-	+
Xcnv64+Xcnv69B (control)	+	-	+	+	+	-	+
Xcnv64+Xcnv69C (patient)	+	-	+	+	+	-	+

Numbers in column headers represent position of the STS on chromosome X in kilobasepairs (kbp), human genome hg19. + = positive PCR reaction,— = negative PCR reaction. Primer sequences are in S3 Table.

**Table 4:** Semen parameters of the men without an X chromosome microdeletion and with an X chromosome microdeletion.

parameter	controls w/o deletion	controls with deletion	infertile w/o deletion	infertile with deletion
**total sperm count (millions)**	234 ± 132 (218)	186 ±102 (163)	25 ± 44 (10)	101 ± 196 (15)
**total motile sperm count (millions)** ^**a**^	172 ± 106 (163) ^b^	123 ± 74 (102) ^b^	15 ± 23 (8)	36 ± 51 (10)
**progressive motility (%)** ^**a**^	60 ± 12 (62) ^b^	53 ± 11 (53) ^b^	32 ± 17 (32)	25 ± 19 (19)
**normal sperm morphology (%)** ^**a**^	12 ± 6 (10)	13 ± 7 (10)	3 ± 2 (2)	3 ± 3 (2)

Both Xcnv64 and Xcnv69 were grouped together. Data are represented as averages ± standard deviations (medians).

^a^ These parameters were calculated without azoospermic subjects.

^b^ There was a difference in progressive motility between the controls without and with an X chromosome microdeletion, Student´s t-test P = 0.0360. These groups also differed in total motile sperm count, Student´s t-test P = 0.0365.

## Discussion

The major limitation of our study is the power for detection of rare genetic associations. Relatively small cohort size can thus explain why we did not detect the least frequent Xcnv67 microdeletion, which was present in 1.1% infertile men of the South European cohort. Xcnv67 microdeletion was present exclusively in the males with abnormal spermatogenesis, and presumably could be causative [14]. On the other hand, we detected in our subjects both Xcnv64 and Xcnv69 microdeletions, including all three subtypes of the Xcnv69 microdeletions, which were associated; but not exclusively connected to abnormal spermatogenesis. However, we did not observe infertility association of these microdeletions in our population sample. Most common microdeletion Xcnv64 reached inverse association in our cohort. This cannot be taken as a final disproval of the association, since our study comprised 5.9x less subjects in the patient group compared to [14].Directly qualitatively comparing the sperm concentration of the patients with the microdeletions Xcnv64 and Xcnv69 there were 13 subjects with azoospermia (13/163, i. e. 7.9%), 40 subjects with low sperm concentration (40/463, i.e. 8.6%) and 5 subjects with normal sperm concentration in the South European cohort; in our study there was one subject with azoospermia (1/23, i.e. 4.3%), 4 subjects with oligozoospermia (with or without asthenoteratozoospermia, 4/81, i.e. 4.9%) and one subject with normal sperm concentration, but asthenozoospermia. Therefore in the Czech cohort, microdeletion frequency in patients appears lower compared to the South European cohort. On the other hand, microdeletion frequency in controls of this study (Xcnv64 15/131, i.e. 11.5%, Xcnv69 6/131, i.e. 4.6%) is strikingly high compared to the population sample of Southern Europe (3.1% and 1.6% for Xcnv64 and Xcnv69 respectively). PCR errors could be a potential source of false positivity, however, the Xcnv64 region failed to amplify even when annealing temperature was lowered down to 53°C or using different DNA polymerase, while other PCR products on X chromosome, in vicinity of the deleted region, were amplified under standard conditions (Table 3). Moreover, Xcnv69 was validated by deletion bridging PCR for types A and B. The type of semen abnormality in Xcnv64 and Xcnv69 carriers corresponds well between the cohorts, given the limits of our cohort size. There is also no substantial difference in the general type of abnormality in the whole patient group (our cohort: 23 azoospermic, 81 oligospermic (with or without asthenozoospermia and teratozoospermia) and 3 asthenozoospermic men; South European cohort: 163 azoospermic, 463 oligospermic men and one with normal sperm concentration, but infertile subject. Ratio of the oligozoospermic to azoospermic patients was not different in our cohort in comparison to the South European cohort (χ^2^ test P = 0.36). This was despite the fact that our study used the 2010 WHO guidelines, while the South European study used less strict 1999 guidelines for sperm concentration.

Stepping aside from the differences in the frequency of the microdeletions, the Xcnv64 and Xcnv69 microdeletions are present in both populations. The reason, why the association was not detected in our study, could also be other factor differentiating these populations. Apart of stochastic effects, these other factors could be genetic differences in other susceptibility alleles that would together with the X chromosome microdeletions contribute to the disease risk in South European population, but such alleles would be less frequent in Czechia. Other possibility being that the microdeletions would form haplotypes containing true susceptibility alleles, again more frequent (or showing higher linkage disequilibrium) in Southern Europe. It is also plausible that due to other factors both genetic and environmental, the effect of the X chromosome microdeletions could be just smaller in the Czech population. This is suggested by the observation of possibly decreased progressive motility and total motile sperm count in the normospermic carriers of the X chromosome microdeletion(s) compared to normospermic men without the microdeletions. In the South European cohort, both total sperm count and total motile sperm count was decreased in Xcnv64 carriers while progressive sperm motility (and total motile sperm count) was decreased in Xcnv69 carriers. However, the effect was fully attributable to the association of the microdeletions with lower semen parameters (and infertility), since there was no significant difference when only control or only infertile subjects with the microdeletion were compared to the subjects of the same group without the microdeletion [14]. Therefore our results might be slightly different, as we observed no detectable association of the microdeletions with infertility, but progressive motility decrease in the control, normospermic subjects carrying the microdeletion.

The microdeletions Xcnv64 and Xcnv69 do not contain any protein-coding gene, and they also do not show high level of sequence conservation, so they may not contain a functional element contributing to normal fertility, making it possible that a true functional element is closely linked.

## Conclusions

In conclusion, association of X-chromosome microdeletions at Xq27.3 and Xq28 with male infertility could not be confirmed nor excluded for Czech males. The data suggest a possibility that the detected microdeletions Xcnv64 and Xcnv69 might be associated with lower semen quality within the normal range. However, these microdeletions do not seem to confer a significant risk of impaired spermatogenesis in the Czech population. The potential clinical role of the previously reported patient-specific Xcnv67 remains to be determined in a larger study population.

## Supporting Information

S1 Table Smoking in the studied population sample. 2x2 contingency table demonstrates smoking to be more prevalent in infertile men compared to controls in our sample. Fisher´s exact test p = 0.040; OR 1.84, 95% CI 1.03 to 3.28. In smokers, there was no difference between infertile men and controls in cigarette number per day (13.3 ± 8.1 vs. 12.4 ± 8.9, P = 0.66).
(DOCX)

S2 Table Semen parameters of X chromosome microdeletion carriers. Evaluation criteria: oligozoospermia = sperm concentration < 15 millions/ml or total sperm count < 39 millions, teratozoospermia = morphology < 4% normal forms, asthenozoospermia = progressive motility < 32%. * evaluation result is known, but the parameters were not disclosed to the authors. ** based on sperm concentration only.
(DOCX)

S3 Table Primers used to amplify sequence tagged sites on chromosome X. (DOCX)
