# Supplementary material for: Recurrent Microdeletions at Xq27.3-Xq28 and Male Infertility: A Study in the Czech Population
Source: PLoS One. 2016 Jun 3;11(6):e0156102. doi: 10.1371/journal.pone.0156102 (PMC4892532; doi:10.1371/journal.pone.0156102)
Supplement: S1 Table — 2x2 contingency table demonstrates smoking to be more prevalent in infertile men compared to controls in our sample. Fisher´s exact test p = 0.040; OR 1.84, 95% CI 1.03 to 3.28. In smokers, there was no difference between infertile men and controls in cigarette number per day (13.3 ± 8.1 vs. 12.4 ± 8.9, P = 0.66). (DOCX) [file pone.0156102.s001.docx]

**S1 Table.** **Smoking in the studied population sample.**

|  | **infertile men** | **controls** | **sum** |
| --- | --- | --- | --- |
| **smokers** | 36 | 32 | 68 |
| **nonsmokers** | 58 | 95 | 153 |
| **sum**^a^ | 94 | 127 | 221 |

2x2 contingency table demonstrates smoking to be more prevalent in infertile men compared to controls in our sample. Fisher´s exact test p = 0.040; OR 1.84, 95 % CI 1.03 to 3.28. In smokers, there was no difference between infertile men and controls in cigarette number per day (13.3 ± 8.1 vs. 12.4 ± 8.9, P = 0.66)

^a^Not all subjects entering the study did state their smoking behavior.
