# Supplementary material for: Recurrent Microdeletions at Xq27.3-Xq28 and Male Infertility: A Study in the Czech Population
Source: PLoS One. 2016 Jun 3;11(6):e0156102. doi: 10.1371/journal.pone.0156102 (PMC4892532; doi:10.1371/journal.pone.0156102)
Supplement: S2 Table — Evaluation criteria: oligozoospermia = sperm concentration < 15 millions/ml or total sperm count < 39 millions, teratozoospermia = morphology < 4% normal forms, asthenozoospermia = progressive motility < 32%. * evaluation result is known, but the parameters were not disclosed to the authors. ** based on sperm concentration only. (DOCX) [file pone.0156102.s002.docx]

**S2 Table. Semen parameters of X chromosome microdeletion carriers.**

| code | X chromosome | sperm concentration (millions/ml) | total sperm count | total motile sperm count | progressive motility (%) | normal forms (%) | evaluation |
| --- | --- | --- | --- | --- | --- | --- | --- |
| C554 | Xcnv64 | 20 | 70 | 46 | 40 | 10 | normospermia |
| N10 | Xcnv64 | 19 | 74 | 59 | 53 | 15 | normospermia |
| N30 | Xcnv64 | 22 | 101 | 55 | 45 | 10 | normospermia |
| N35 | Xcnv64 | 22 | 46 | 32 | 45 | 20 | normospermia |
| N62 | Xcnv64 | 43 | 163 | 76 | 33 | 8 | normospermia |
| N102 | Xcnv69typeA | 31 | 130 | 84 | 52 | 4 | normospermia |
| C679 | Xcnv64 | 49 | 250 | 158 | 49 | 5 | normospermia |
| N21 | Xcnv64 | 50 | 100 | 60 | 50 | 25 | normospermia |
| N64 | Xcnv69typeA | 70 | 294 | 126 | 36 | 10 | normospermia |
| N81 | Xcnv69typeA | 50 | 160 | 128 | 60 | 30 | normospermia |
| N115 | Xcnv64 | 84 | 168 | 102 | 54 | 10 | normospermia |
| C637 | Xcnv69typeA | 80 | 224 | 168 | 63 | 10 | normospermia |
| N63 | Xcnv64 | 70 | 329 | 259 | 71 | 20 | normospermia |
| N101 | Xcnv64 | 100 | 380 | 243 | 53 | 7 | normospermia |
| N117 | Xcnv69typeB | 100 | 240 | 168 | 60 | 10 | normospermia |
| N50 | Xcnv64+69typeB | 100 | 110 | 77 | 70 | 20 | normospermia |
| N119 | Xcnv64 | 111 | 322 | 249 | 66 | 10 | normospermia |
| C571 | Xcnv64 |  |  |  |  |  | normospermia^a^ |
| N41 | Xcnv64 |  |  |  |  |  | normospermia^a^ |
| N90 | Xcnv64 |  |  |  |  |  | normospermia^a^ |
| Y473 | Xcnv69typeB | 0 | 0 | 0 | 0 | 0 | azoospermia |
| Y450 | Xcnv64+69typeC | 1,1 | 5 | 1 | 7 | 1 | oligoasthenoteratozoospermia |
| Y459 | Xcnv69typeA | 2,3 | 11 | 2 | 10 | 1 | oligoasthenoteratozoospermia |
| Y572 | Xcnv64 | 5,8 | 20 | 10 | 33 | 2 | oligoasthenoteratozoospermia |
| Y495 | Xcnv64 | 13 | 70 | 49 | 54 | 7 | oligozoospermia^b^ |
| Y458 | Xcnv64 | 79 | 498 | 120 | 19 | 4 | asthenozoospermia |

Evaluation criteria: oligozoospermia = sperm concentration < 15 millions/ml or total sperm count < 39 millions, teratozoospermia = morphology < 4% normal forms, asthenozoospermia = progressive motility < 32%.

^a^ evaluation result is known, but the parameters were not disclosed to the authors.

^b^ based on sperm concentration only.
