# Supplementary material for: Recurrent Microdeletions at Xq27.3-Xq28 and Male Infertility: A Study in the Czech Population
Source: PLoS One. 2016 Jun 3;11(6):e0156102. doi: 10.1371/journal.pone.0156102 (PMC4892532; doi:10.1371/journal.pone.0156102)
Supplement: S3 Table — (DOCX) [file pone.0156102.s003.docx]

**S3 Table**. **Primers used to amplify sequence tagged sites on chromosome X.**

| STS name | forward primer | reverse primer | chromosome position (hg19) | expected product size |
| --- | --- | --- | --- | --- |
| X143.624 | tgatcctgtgccttgaaatg | cacctgccttgtacccacat | chrX:143624116-143624314 | 199 |
| X143.640 | tcatgacagccttcacagaaa | gctcaggattcctctggcta | chrX:143640541-143640670 | 130 |
| X154.366 | TCGTGCTAATGTGCATGTGTT | TATGTGGCCTGCTGAGAATG | chrX:154366488-154366616 | 129 |
| X154.417 | ggtcacagttagcgctcaaaa | ATATTAGTGGGCGGGAGGAC | chrX:154417534-154417632 | 99 |
